# Similar Inflammatory Adaptation in Women following 10 Weeks of Two Equalized Resistance Training with Different Muscle Action Duration

**DOI:** 10.1155/2022/2185111

**Published:** 2022-06-15

**Authors:** Kelerson Mauro de Castro Pinto, Rodrigo César Ribeiro Diniz, Frank Douglas Tourino, Lucas Túlio de Lacerda, Eduardo Bearzoti, Karine Beatriz Costa, Débora Maria Soares de Souza, Fernando Vitor Lima, Etel Rocha-Vieira, Mauro Heleno Chagas, Andre Talvani

**Affiliations:** ^1^Escola de Educação Física, Laboratório de Fisiologia do Exercício, Universidade Federal de Ouro Preto (UFOP), Ouro Preto, Minas Gerais, Brazil; ^2^Departamento de Ciências Biológicas-Laboratório de Imunobiologia da Inflamação, Universidade Federal de Ouro Preto (UFOP), Ouro Preto, Minas Gerais, Brazil; ^3^Universidade Federal de Minas Gerais (UFMG), Escola de Educação Física, Fisioterapia e Terapia Ocupacional, Laboratório do Treinamento na Musculação, Belo Horizonte, Minas Gerais, Brazil; ^4^Pontifícia Universidade Católica de Minas Gerais (PUC-MG), Departamento de Educação Física, Belo Horizonte, Minas Gerais, Brazil; ^5^Universidade do Estado de Minas Gerais, Divinópolis, Minas Gerais, Brazil; ^6^Departamento de Estatística, Universidade Federal de Ouro Preto (UFOP), Ouro Preto, Minas Gerais, Brazil; ^7^Programa Multicêntrico de Pós-Graduação em Ciências Fisiológicas, Sociedade Brasileira de Fisiologia, Universidade Federal dos Vales do Jequitinhonha e Mucuri (UFVJM), Diamantina, Brazil

## Abstract

This study is aimed at evaluating the profile of inflammatory markers and components of redox regulation in untrained women after 10 weeks of resistance training using equalized protocols but different muscle action duration (MAD). Twenty-two women underwent progressive resistance training exercising the knee extensor muscles for 10 weeks—3x/week, with 3-5 sets of 6 repetitions at 50% of the 1 repetition maximum strength test (1RM), with a rest of 180 s between the series, following the training protocol (i) 5 s of concentric muscle action for 1 s of eccentric muscle action (5C-1E) and (ii) 1 s of concentric muscle action for 5 s of eccentric muscle action (1C-5E). Quadriceps muscle hypertrophy maximum strength (1RM) and redox regulation/muscle damage/inflammatory markers (CAT, SOD, TBARS, FRAP, CH, LDH, CXCL8, and CCL2) were evaluated. Plasma markers were evaluated before and 30 minutes after the first and last training sessions. A similar gain in hypertrophy and maximum strength was observed in both groups. However, in the 5C-1E, a significant major effect was observed for SOD (*F*_1.19_ = 10.480, *p* = 0.004) and a significant major time effect, with a reduction in the last training session, was observed for CXCL8 (*F*_1.37_ = 27.440, *p* < 0.001). In conclusion, similar protocols of resistance training, with different MAD, produced similar inflammatory and adaptive responses to strength training. As the training load is progressive, the maintenance of this inflammatory and redox regulation profile suggests an adaptive response to the proposed strength training.

## 1. Introduction

Several studies have investigated the acute and chronic effects of resistance training modulating the inflammatory and redox profiles [1–6]. Such effects can contribute to a better understanding of the role of resistance training in the prevention and control of some diseases, as well as in the adaptive process itself, which is mainly associated with muscle hypertrophy [1, 2, 4, 6].

After a training session, the inflammatory response, together with other mechanisms, is responsible for the regeneration and repair of injured cells, as well as for processes related to muscle hypertrophy [6–8]. Chemokines, which are mainly associated with monocytes/macrophages and neutrophils, such as CCL2, CCL3, CCL5, and CXCL8 [8], and the increase of antioxidant capacity [5], seem to have a fundamental role in the process of regeneration/hypertrophy of muscle tissue improving.

However, the effect of resistance training on the inflammatory profile and redox regulation is still controversial. Some studies investigated different biomarkers and training methods while others did not equalize the training protocols and did not monitor repetition duration [3, 4, 9]. Thus, the equalization of training protocols will provide more accurate results compared to resistance training protocols.

It is known that one of the variables that influences the adaptations of resistance training is repetition duration [10]. Different eccentric (ECC) and concentric (CON) MAD can be observed for the same repetition duration. Gillies et al. [11] demonstrated that the group with the longest of the CON action duration (6 s CON-2 s EXC) had significant hypertrophy in type I and type II fibers, while the protocol group, with the longest of the EXC action duration (2 s CON-6 s EXC), showed hypertrophy only in type I fibers. However, there were no differences between groups in each type of fiber. Moreover, the performance of both groups increased similarly in the 1RM test in the inclined leg press exercise.

Therefore, controlling MAD becomes essential to ensure that any differences observed in the comparison of different equivalent training protocols, varying only the of muscle actions duration, are not due to the manipulation of the total time under tension. Some authors have reported that, even in protocols equalized by the volume of training, the repetition duration can directly influence acute responses [12] and chronicle responses of strength training [10, 13]. Furthermore, Goto et al. [14], when comparing the acute response in resistance training protocols, with the same repetition duration and different combinations of CON and EXC muscle actions, observed a greater metabolic response for the protocol with longer CON duration. Although the authors reported using a metronome to control the muscle actions duration, they did not report the duration of these actions. Gillies et al. [11] also reported using a metronome to control the muscle action duration and did not report the duration of muscle actions and repetitions. Moreover, these authors have followed the progression of intensity by monitoring an exercise (i.e., leg press); they did not report the values of the three other exercises used in the training protocol. To overcome some of the reported limitations, this study monitored and recorded the muscle action duration and used a single exercise, facilitating control over the equalization of the training load associated with the protocol.

Therefore, this study is aimed at evaluating the profile of chemokines and redox regulation in untrained women before and after 10 weeks of resistance training in response to similar protocols but with different muscle action duration. It was hypothesized that in both protocols, changes in acute responses would be observed in the studied biomarkers, mainly with a longer CON muscle action duration, in an equalized protocol, controlling and recording the muscle action duration. Furthermore, greater responses of hypertrophy and muscle strength were also expected, especially for the protocol with longer duration of CON muscle action, in addition to an increase in antioxidant responses and reduced chemokine response as an adaptation to strength training.

## 2. Methods

### 2.1. Participants

Twenty-two untrained volunteers that matched the following criteria were enrolled in this study: (1) young female (18 to 35 years old) in oral contraceptive use, (2) no cigarette smoking, and (3) absence of lower limb, spinal, and pelvic musculoskeletal lesions in the six months prior to the study. Volunteers were previously interviewed and related do not practice strength exercise in the last six months. Most of them practiced aerobic exercise (walking), less than 3x per week, and they were distributed between groups 1 and 2 of the study. The sample size calculation was performed with the G∗Power for Windows version 3.1.9.2 (Düsseldorf, Germany) according to guidelines proposed by Beck [15]. The a priori statistical power (1 − *β*) of 0.8 and 5% significance level were adopted. The study was approved by the local ethics committee (CAAE 30594714.0.1001.5149) and carried out in accordance to the Declaration of Helsinki.

### 2.2. Procedures

In the first session, a magnetic resonance imaging exam was performed to assess muscle cross-sectional area (CSA). After an interval of 0 to 48 h, one repetition maximum (1RM) test was performed in the second (familiarization) and third (pretest) experimental sessions. After that, participants engaged the training intervention differentiated only by muscle action duration (30 sessions within 10 weeks). A trained clinical pathologist technician collected 10 mL of venous blood in the first and last training sessions before and 30 min after the end of the training exercise, in a private and climatized room at UFMG. Blood was collected in ethylenediaminetetraacetic acid (EDTA) tubes (BD Vacutainer, Franklin Lakes, NJ, USA) centrifuged for 10 minutes at 1,500 × *g* in a refrigerated centrifuge (Thermo Scientific, Sorvall X4 Pro). Plasma and erythrocytes, independently, were collected and stored at -80°C for further analysis. Magnetic resonance imaging was repeated for all groups 72-120 h after the last training session, followed by the 1RM test.

### 2.3. 1RM Testing and Training Protocols

The 1RM test and training sessions were performed on a seated knee extension machine. The 1RM test was determined in the CON mode with a maximum of six attempts and 180 s rest interval was given between attempts [16]. The volunteer was asked to perform the hard and fast force as possible, trying to reach the 30° angle of knee extension (0° = full knee extension). Thus, the 1RM value corresponded to the weight lifted in the previous successful attempt. Based on the mean value of 1RM test between the familiarization and pretest sessions, the following intersession reliable values were obtained: intraclass correlation coefficient (ICC_[3, 1]_) = 0.98; standard error of measurement = 3.07 kg; relative standard error of measurement = 8.9%.

All individuals performed different resistance training protocols for 10 weeks (3 times week with 48-72 h recovery between sessions) and attended all the scheduled sessions. Trainings occurred in different period of the day; however, each woman kept her specific time to perform this training for 10 weeks. Our decision to perform 10 weeks of training was based on previous studies whose muscle hypertrophy in men and women started at 6 and obtained its higher performance around 10 weeks of strength exercises [17–19].

Generally, all protocols consisted of 3-5 sets (3 sets during weeks 1-2; 4 sets during weeks 3-4; 5 sets during weeks 5-10) of 6 repetitions at 50% of 1RM, 180 s rest between sets and 6 s duration of the repetition. These training loads were based on the prescription of Tanimoto & Ishii [10] and adjusted following a pilot study (reducing the number of repetitions and increasing the pause) to guarantee the feasibility of training protocols which should be matched. However, experimental groups were differentiated by different MAD: 5 s of concentric muscle action for and 1 s of eccentric muscle action (group 5C-1E) and 1 s of concentric muscle action for 5 s of eccentric muscle action (group 1C-5E) (Table 1).

To control the variables related to the proposed training protocol, the seated knee extension machine was adapted to give access to muscle action duration and range of motion. A potentiometer with 10 k*Ω* was fixed in the rotation axis of the fixed lever of the knee extensor machine. The potentiometer data was used to generate angle versus time curves and, hence, to determine the range of motion and MAD during the training sessions. Table 1 shows the proposed training protocol and the protocol that was performed in each group.

At every two weeks, in the 7^th^, 13^th^, 19^th^, and 26^th^ training sessions, 1RM test was performed before starting the training session. These procedures are aimed at maintaining the relative intensity (50% 1RM) within the proposed training protocol settings throughout 10 weeks of training. A 10-minute rest period was established between the 1RM test and the start of the training session. A metronome (auditive feedback) and potentiometer data (visual feedback) were used to guarantee MAD and range of motion throughout the set.

### 2.4. Muscular Cross-Sectional Area

**Figure 1:**
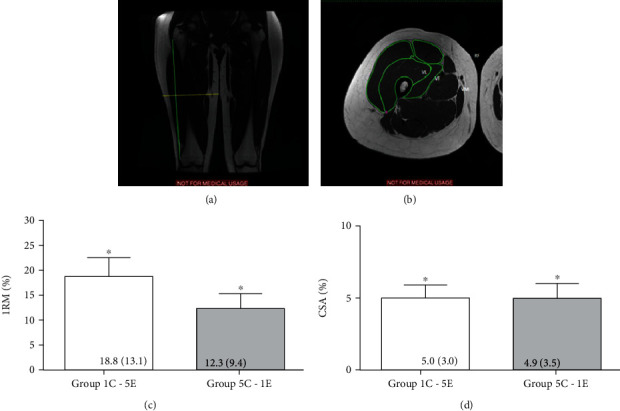
Reference for evaluation of quadriceps magnetic resonance image (MRI) and resistance training results. (a) Image obtained from the MRI of one of the volunteers, for reference of positioning of the cut points. The vertical line measures the size of the femur, and the horizontal line represents 50% of the distance between the upper edge of the patella and the anterior superior iliac spine. (b) Image obtained from the MRI of one of the volunteers. Cut to 50% of the distance between the upper edge of the patella and the anterior superior iliac spine. The marking was performed through the Osirix 6.0 software to calculate the transverse section area of the rectus femoris (RF), vastus lateralis (VL), vastus intermedius (VI), and vastus medialis (VM) muscles. Percent variation of performance in the test of (c) one-repetition maximum (1RM) and of the (d) cross-sectional area (CSA) in the quadriceps muscle, for the training protocols: group 1C-5E (1 s of concentric muscle action and 5 s of eccentric muscle action) and group 5C-1E (5 s of concentric muscle action and 1 s of eccentric muscle action). ^∗^*p* < 0.0001 = significant increase compared to measurements taken preexercise.

Measurements of body mass, height, and fat percentage were performed. Fat percentage was evaluated using the skinfold technique, according to the protocol used by Jackson and Pollock [20]. After the initial evaluation, to assess muscle cross-section area, participants were taken to the magnetic resonance image exam at the Ecoar® Diagnostic Imaging Clinic (Belo Horizonte, MG, Brazil). Participants were lying on a stretcher with their muscles relaxed for at least 20 minutes before the tests began. The magnetic resonance image recording was performed on a Sigma HDX 1.5 Tesla device (GE Medical System, USA) with a repetition time of 600 ms, echo time of 8.4 ms, a slice thickness of 6 mm, interslice gap of 0.6 mm, field of view of 240 mm, and resolution of 320 × 256 pixels. The coronal and axial images were taken between the major trochanter and the lateral epicondyle of the femur on the right thigh. All images were stored for offline analysis by two evaluators blinded for the treatments. The distance between the trochanter major and epicondyle lateral of the femur (femur length) was determined by the coronal images of the Osirix 6.0 software. Axial slice images at 50% of the femur length were used to measure CSA of rectus femoris (RF_CSA_), vastus medialis (VM_CSA_), vastus lateralis (VL_CSA_), and vastus intermedius (VI_CSA_) for each participant. The sum of the areas of these muscles was considered the CSA of quadriceps femoris (QF_CSA_) (Figures 1(a) and 1(b)).

To minimize a possible modification on the image position between pre- and posttests, extra images (one right above and right below image) from the reference slice point were also analyzed. The mean of 3 images was analyzed for QF. Based on the mean value of the 3 CSA, the following interrater reliable values were obtained: ICC = 0.98; standard error of measurement = 1.4 cm^2^; relative standard error of measurement = 2.8%.

### 2.5. Blood Collection and Chemokine Immunoassays

Circulating levels of CCL2 and CXCL8 (PeproTech, USA) were detected in plasma previously stored at -80°C. These biomarkers were measured, in duplicate, by enzyme-linked immunosorbent assay and following the manufacturers' recommendations. The absorbance reading was performed on a microplate reader (SpectraMax® 190, Molecular Devices, CA, USA), using 450/630 nm ratio of wavelength.

### 2.6. Plasma Redox Analysis

To evaluate the blood redox marker enzymes of redox regulation, lysis of erythrocyte was performed according to Glass and Gershon [21]. The supernatant of the erythrocyte lysate was used to analyze the total protein content [22], the thiobarbituric acid reactive substances (TBARS) [23], total antioxidant capacity by the ferric reducing ability of plasma (FRAP) [24], and the activities of superoxide dismutase (SOD) [25] and catalase (CAT) [26]. The absorbance reading was performed on a microplate reader (SpectraMax® 190, Molecular Devices, CA, USA), according to the recommended wavelength from each manufacturer.

### 2.7. Muscle Injury Biomarkers

The activity of the lactate dehydrogenase (LDH) and creatine kinase (CK) were measured using the kinetic method (LDH Liquiform and CK-NAC Liquiform, Labtest), by an Olympus AU640 autoanalyzer (Olympus, Hamburg, Germany) using the manufacturer's appropriate reagents. Calibration and quality control of the equipment were also performed according to the recommended protocol.

### 2.8. Statistical Analysis

Initially, age, body mass, height, fat percentage, and 1RM means at the beginning of the trial were compared with *t*-tests, to confirm that there were no differences between the two groups of volunteers assigned to each training protocol. Training protocols were compared by means of a two-way ANOVA model with repeated measurements (factor 1 = group; factor 2 = time) and variables transformed into relative responses: (posttest − pretest)/pretest∗100. Models were fitted using the “Proc Mixed” of the SAS software. A residual analysis was then performed, and normality was verified with the Shapiro-Wilk test. Whenever normality assumption did not hold, a search for outliers was done, discarding observations with standardized residues higher (in absolute value) than what would be expected under normality, given the total number of observations. For some variables, outlier discarding was not sufficient to attain normality, and so Box and Cox [27] transformation was used. Finally, type 3 *F*-tests were carried out to test the significance of the factors of the model. Type 3 *F*-tests are suitable when data sets are unbalanced and correspond to the usual *F*-test whenever data are balanced. There was no need of post hoc tests since each factor had only two levels each. Effect sizes were calculated as Cohen's *d* values to further examine the magnitude of acute change in all variables from pre- to postexercise. These values are reported to reflect the magnitude of the differences in each treatment where ≤0.20 was considered “trivial,” 0.21-0.49 “small,” 0.50-0.79 “moderate,” and ≥0.80 “large.”

## 3. Results

The volunteers were equally distributed according to the result of the 1RM test, and there was no statistically significant difference between the protocols for 1RM results (*p* = 0.670) and for other sample characterization parameters before beginning the training period (body mass *p* = 0.0.653 and body fat *p* = 0.518). In group 1C-5E, volunteers used contraceptives for an average of 2.7 ± 1.4 years, with one volunteer using single-phase combined contraceptives, 9 using two-phase combined contraceptives, and one using injectable contraceptives. The mean time of use in group 5C-1E was 2.6 ± 1.8 years, with one volunteer using single-phase combined contraceptives and 10 volunteers using two-phase combined contraceptives.

After 10 weeks of training, although the two-way analysis of variance for the 1RM test did not show significant interaction (time × group, *F*_1.21_ = 3,370, *p* = 0.08), a significant main effect was observed for time (*F*_1.21_ = 6,540, *p* < 0.001). There was an increase in performance, in percentage, and in the 1RM test for both groups: 1C-5E (18.8 ± 13.1%; *d* = 0.50) and 5C-1E (12.3 ± 9.6%; *d* = 0.95) (Figure 1(c)) when compared with the pretraining. The CSA of the quadriceps muscle (*F*_1.20_ = 0.030, *p* = 0.856), when compared with the pretraining also presented an increase main effect for time (*F*_1.20_ = 70.430, *p* < 0.001). 5.3% ± 3.0% (*d* = 0.39) and 4.9 ± 3.5% (*d* = 0.34) are for the groups 1C-5E and 5C-1E, respectively (Figure 1(d)). However, no differences were observed to the 1RM (Figure 1(c)) and CSA (Figure 1(d)) concerning both training protocols. No significant interaction was observed in the activity of CK and LDH enzymes (time × group: *F*_1.12_ = 0.030 and *p* = 0.865 and *F*_1.18_ = 0.010 and *p* = 0.934, respectively), and no significant main effects were observed for time and training protocols.

**Figure 2:**
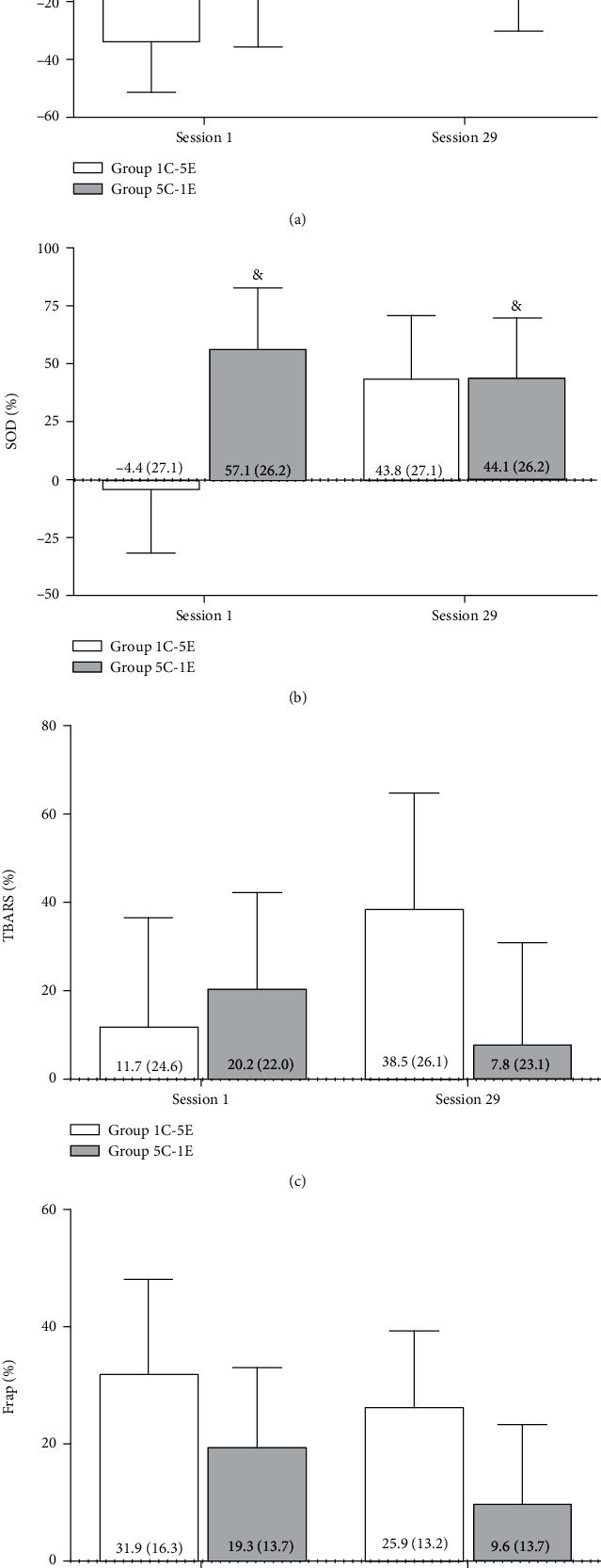
Percentage variation, at the beginning and after 10 weeks of resistance training, for (a) catalase (CAT), (b) superoxide dismutase (SOD), (c) thiobarbituric acid reactive substances (TBARS), and (d) ferric reducing ability of plasma (FRAP); for the training protocols: group 1C-5E (1 s of concentric muscle action and 5 s of eccentric muscle action) and group 5C-1E (5 s of concentric muscle action and 1 s of eccentric muscle action). & = difference between the two resistance training protocols (*p* = 0.004).

Although no significant interaction effect was observed in the analysis of SOD activity (time × group, *F*_1.19_ = 0.090, *p* = 0.772), here was a significant main group effect (*F*_1.19_ = 10.480, *p* = 0.004), with a greater percentage change being observed in group 5C-1E (Figure 2(b)). No significant interaction and main effects were observed for the other redox status markers (CAT, TBARS, and FRAP) (Figures 2(a), 2(c), and 2(d)).

**Figure 3:**
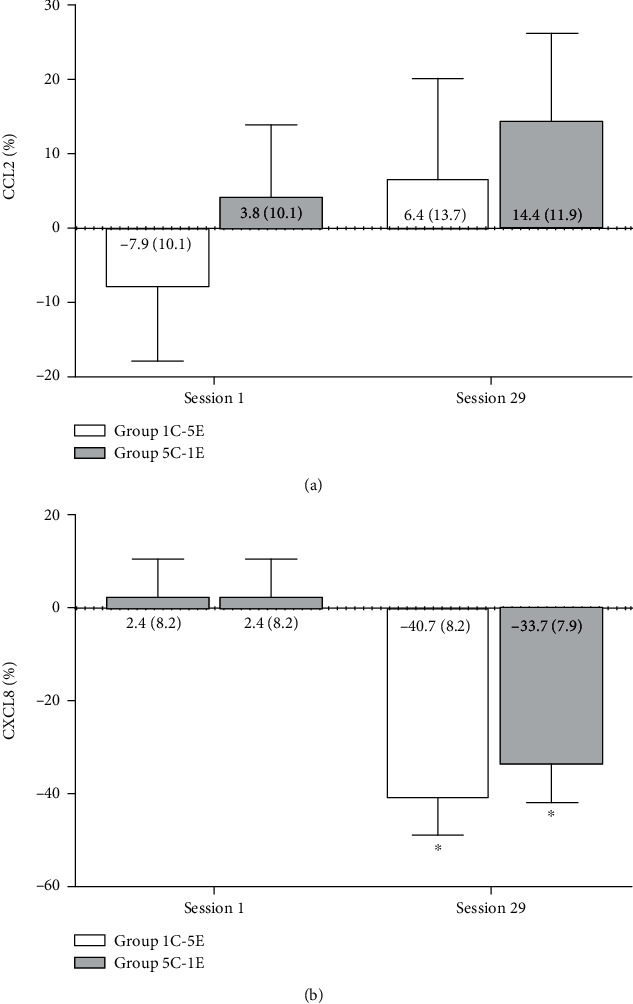
Percent change, at the beginning and after 10 weeks of resistance training, for the chemokines (a) CCL2 and (b) CXCL8, for the training protocols: group 1C-5E (1 s of concentric muscle action and 5 s of eccentric muscle action) and group 5C-1E (5 s of concentric muscle action and 1 s of eccentric muscle action). ∗ = difference between the two training sessions (*p* < 0.001).

No significant interaction effect of chemokines was observed on the CCL2 profile (time × group, *F*_1.32_ = 0.030, *p* = 0.870), as well as for the main effects of time and group (time: *F*_1.32_ = 1.160, *p* = 0.289; group: *F*_1.32_ = 0.730, *p* = 0.400) (Figure 3(a)). In CXCL8, although there were no significant interaction effects (time × group, *F*_1.37_ = 0.570, *p* = 0.455) and group effects (group, *F*_1.37_ = 0.490, *p* = 0.488), a significant main effect of time was observed with reduction in the 29^th^ training session (*F*_1.37_ = 27.440, *p* < 0.001) (Figure 3(b)).

## 4. Discussion

This study evaluated the inflammatory and redox regulation profile of untrained women, before and after 10 weeks of resistance training, in response to equalized protocols but with different MAD. The main findings of this study show that inflammatory responses and redox regulation are similar between equalized training protocols, and this profile remained unchanged after 10 weeks of training. Moreover, protocols with different MAD did not result in different muscle hypertrophy and strength responses.

To ensure that any observed effect, both on muscle hypertrophy and on inflammatory and redox responses, was due to the different MAD, the training protocols remained equalized in the number of sets, repetitions, repetition duration, relative intensity of the exercise (adjusted at the same times and based on the results of the 1RM tests), range of motion, and rest interval. It should be noted that visual and auditory stimuli were also provided to assist in controlling the MAD, which were recorded and analyzed during training sessions. Then, while Gillies et al. [11] established the muscle action's duration using a metrometer, in this present study performed, this same variable was evaluated using a potentiometer. The potentiometer registered each muscle action and movement amplitude, allowing to affirm that our data occurred in detriment of different MAD between groups.

In this study, both protocols promoted adaptations of strength and muscle hypertrophy without, however, showing differences between them. These results do not corroborate the findings of Gillies et al. [11] who reported different responses in the parameters used for muscle hypertrophy in protocols with different MAD. Despite also using resistance training protocols with the same MAD and different repetition durations, the study of Chazaud [28] uses 6-8 RM ranges in the configuration of the training protocols. The application of these bands can cause a variation in intensity between groups, as MAD influences the maximum number of repetitions that can be performed for the same relative intensity [14]. These differences in the configuration of the training load may have impaired the equalization of the protocols, leading to greater metabolic stress, as observed by the higher concentrations of cortisol, thus explaining the different results of hypertrophy for the group with longer duration of CON action. Moreover, because muscle hypertrophy does not occur homogeneously between the quadriceps femoris muscles [29], the different exercises for lower limbs used in the study of Gillies et al. [11] could also have contributed to the different results [30], mainly because muscle hypertrophy was only analyzed in one region of the vastus lateralis.

As with muscle hypertrophy, maximum strength gain was similar for both training protocols. This finding is supported by data from Guillies et al. [11]. The increase in maximum strength is caused by the CSA and neural factors [31]. As there were no differences in CSA between the two groups, there would also be no difference in maximum strength performance. Although experimental design of this study does not allow us to make inferences about the neural mechanisms of strength control, it is believed that any adaptation produced by the training protocols has similarly influenced strength production.

As previously mentioned, studies that compared different training strategies showed methodological gaps that may interfere with the interpretation of the results [3, 9]. The phenotype of immune cells as well as the set of inflammatory mediators released during resistance exercise might define the inflammatory environmental inside the muscle [3]. In this study, similar inflammatory responses (CK, LDH, and chemokines) and redox status can be attributed to the equality of training protocols. Thus, for the studied sample, the impact of different MAD may not have been enough to produce metabolic and endocrine changes or even changes resulting from muscle damage that would lead to an inflammatory profile and different redox regulation between the proposed protocols. This is because all the other variables in the training protocols were equalized.

Although there was no difference in the inflammatory responses (CK, LDH, CCL2, and CXCL8) and the redox regulation in the training protocols, there were variations in the responses of these parameters as a function of the resistance training. These results corroborate other studies that demonstrated that resistance training can produce changes in inflammation and redox regulation [1, 4, 32, 33].

Studies have demonstrated the importance of inflammatory responses in muscle regeneration and hypertrophy ([8, 34],), as well as in the relationship between inflammatory response and redox balance [5, 6]. Moreover, several authors found differences in tissue and plasma responses to cytokines after exercise [35, 36], thus suggesting that the absence of plasma inflammatory marker overproduction does not necessarily imply their nonparticipation in the adaptation to training.

Regarding the adaptations that resistance training can produce in inflammatory responses and redox regulation, some studies have shown improvement in the antioxidant capacity and the inflammatory profile [3, 4]. The lack of difference in the response variation in the last training session and the first session, in a training with progressive load, suggests that the proposed resistance training produced adaptations in inflammation and the redox regulation. This is because the same response was observed for a greater training load.

Although neutrophils are the most populous cells at the beginning of the local inflammatory process, the response of CXCL8 reduced in the last exercise session, which corroborates with Hirose [37]. Studies have demonstrated a wide variation in the response of this chemokine in different types of exercise, such as increased plasma concentration after long-term aerobic exercises ([38],), no change, or even a reduction after a resistance exercise [37, 39]. This variable behavior would be associated with the changes produced by different types of exercise (metabolic, endocrine changes, etc.), as well as by the chemokine function itself (angiogenesis, neutrophil chemotaxis, etc.). Furthermore, some authors suggest that the plasma response does not always result from tissue changes, suggesting a paracrine effect, especially for resistance exercises [35, 37].

Even with the methodological precautions taken, this study has some limitations. Firstly, because it analyzed hypertrophy in only one region of the quadriceps muscle, other portions of the muscle may have shown different responses. This aspect is reinforced by studies that identified regional muscle hypertrophy after using different training protocols [18, 40]. Moreover, the volunteers were not provided with a diet, being only asked to maintain the same dietary pattern 24 hours before the training sessions with blood collection, and as this study uses a single monoarticular exercise, its results must be interpreted with caution when applied to other training proposals.

## 5. Conclusion

The results show that the equalized strength training protocols proposed in this study, with different duration of muscle actions and applied in untrained women, produced responses from the analyzed chemokines and similar redox regulation, and this profile remained unchanged after 10 weeks of training. As the training load is progressive, with increased volume, maintaining this profile of chemokines and redox regulation would suggest an adaptive response to the proposed strength training. Finally, based on these data, physical trainers can obtain consistent responses related to the femoral quadriceps hypertrophy and increase of maximum strength in the knee extension applying combined training protocols with different duration of muscle actions. However, further investigations concerning inflammatory responses using both training protocols should be performed to a global comprehension of the muscle hypertrophic process.

## Figures and Tables

**Table 1:** The proposed training protocol and protocol that was actually performed in each group.

	Training protocol	
	5C-1E	1C-5E
Scheduled		
Set × repetitions	3–5 × 6	3–5 × 6
Intensity (%1RM)	50	50
Concentric muscle action duration (s)	5	1
Eccentric muscle action duration (s)	1	5
Range of motion of the knee (°)	70	70
Actually performed		
Concentric muscle action duration (s)	4.8 ± 0.3	1.1 ± 0.2
Eccentric muscle action duration (s)	1.1 ± 0.2	4.9 ± 0.3
Range of motion of the knee (°)	71.3 ± 3.0	76.4 ± 7.3
Total volume load (kg)	16,013.3 ± 3,247.6	16,507.9 ± 2,637.1

Volume load = sum of all sessions (number of total repetitions in the session∗load of session); % 1RM: percentual of performance of one maximum repetition test; 5C-1E: training protocol with 5 s concentric muscle action and 1 s of eccentric; 1C-5E: training protocol with 1 s concentric muscle action and 5 s of eccentric.

## Data Availability

The biological data used to support the findings of this study are available from the corresponding author upon request.

## References

[1] Chi C. P., Hou C. W., Wu Y. Y., Wang T. H., Yu S. H. (2020). Night time resistance exercise alters muscular IL-6-related protein signaling, but not muscle growth after 10 weeks of resistance training in male rats. *General Physiology and Biophysics*.

[2] Coelho D. B., Lopes L. M. P., Oliveira E. C. (2021). Baseline diet quality is related to changes in the body composition and inflammatory markers: an intervention study based on resistance training and nutritional advice. *BioMed Research International*.

[3] Ihalainen J. K., Ahtiainen J. P., Walker S. (2017). Resistance training status modifies inflammatory response to explosive and hypertrophic resistance exercise bouts. *Journal of Physiology and Biochemistry*.

[4] Lopes L. M. P., Oliveira E. C., Becker L. K. (2020). Resistance training associated with dietetic advice reduces inflammatory biomarkers in the elderly. *BioMed Research International*.

[5] Scheffer D. L., Silva L. A., Tromm C. B. (2012). Impact of different resistance training protocols on muscular oxidative stress parameters. *Applied Physiology, Nutrition, and Metabolism*.

[6] Youm T. H., Woo S. H., Kwon E. S., Park S. S. (2019). NADPH oxidase 4 contributes to myoblast fusion and skeletal muscle regeneration. *Oxidative medicine and cellular longevity 2019*.

[7] Jensen S. M., Bechshøft C. J. L., Heisterberg M. F. (2020). Macrophage subpopulations and the acute inflammatory response of elderly human skeletal muscle to physiological resistance exercise. *Frontiers in Physiology*.

[8] Lu H., Huang D., Ransohoff R. M., Zhou L. (2011). Acute skeletal muscle injury: CCL2 expression by both monocytes and injured muscle is required for repair. *The FASEB Journal*.

[9] González-Badillo J. J., Rodríguez-Rosell D., Sánchez-Medina L., Gorostiaga E. M., Pareja-Blanco F. (2014). Maximal intended velocity training induces greater gains in bench press performance than deliberately slower half-velocity training. *European Journal of Sport Science*.

[10] Tanimoto M., Ishii N. (2006). Effects of low-intensity resistance exercise with slow movement and tonic force generation on muscular function in young men. *Journal of Applied Physiology*.

[11] Gillies E. M., Putman C. T., Bell G. J. (2006). The effect of varying the time of concentric and eccentric muscle actions during resistance training on skeletal muscle adaptations in women. *European Journal of Applied Physiology*.

[12] Martins-Costa H. C., Diniz R. C. R., Lima F. V. (2016). Longer repetition duration increases muscle activation and blood lactate response in matched resistance training protocols. *Revista de Educação Física*.

[13] Tanimoto M., Sanada K., Yamamoto K. (2008). Effects of whole-body low-intensity resistance training with slow movement and tonic force generation on muscular size and strength in young men. *The Journal of Strength & Conditioning Research*.

[14] Goto K., Ishii N., Kizuka T., Kraemer R. R., Honda Y., Takamatsu K. (2009). Hormonal and metabolic responses to slow movement resistance exercise with different durations of concentric and eccentric actions. *European Journal of Applied Physiology*.

[15] Beck T. W. (2013). The importance of a priori sample size estimation in strength and conditioning research. *The Journal of Strength & Conditioning Research*.

[16] Diniz R. C. R., Martins-Costa H. C., Machado S. C., Lima F. V., Chagas M. H. (2014). Repetition duration influences ratings of perceived exertion. *Perceptual and Motor Skills*.

[17] Damas F., Libardi C. A., Ugrinowitsch C. (2018). The development of skeletal muscle hypertrophy through resistance training: the role of muscle damage and muscle protein synthesis. *European Journal of Applied Physiology*.

[18] Diniz R. C. R., Tourino F. D., Lacerda L. T. (2020). Does the muscle action duration induce different regional muscle hypertrophy in matched resistance training protocols?. *Journal of Strength and Conditioning Research*.

[19] Melnyk J. A., Rogers M. A., Hurley B. F. (2009). Effects of strength training and detraining on regional muscle in young and older men and women. *European Journal of Applied Physiology*.

[20] Jackson A. S., Pollock M. L. (1978). Generalized equations for predicting body density of men. *British Journal of Nutrition*.

[21] Glass G. A., Gershon D. (1981). Enzymatic changes in rat erythrocytes with increasing cell and donor age: loss of superoxide dismutase activity associated with increases in catalytically defective forms. *Biochemical and Biophysical Research Communications*.

[22] Bradford M. M. (1976). A rapid and sensitive method for the quantitation of microgram quantities of protein utilizing the principle of protein-dye binding. *Analytical Biochemistry*.

[23] Ohkawa H., Ohishi N., Yagi K. (1979). Assay for lipid peroxides in animal tissues by thiobarbituric acid reaction. *Analytical Biochemistry*.

[24] Benzie I. F. F., Strain J. J. (1996). The ferric reducing ability of plasma (FRAP) as a measure of antioxidant power the FRAP assay. *Analytical Biochemistry*.

[25] Marklund S., Marklund G. (1974). Involvement of the superoxide anion radical in the autoxidation of pyrogallol and a convenient assay for superoxide dismutase. *European Journal of Biochemistry*.

[26] Nelson D. P., Kiesow L. A. (1972). Enthalpy of decomposition of hydrogen peroxide by catalase at 25° C (with molar extinction coefficients of H_2_O_2_ solutions in the UV). *Analytical Biochemistry*.

[27] Box G. E. P., Cox D. R. (1964). An analysis of transformations. *Journal of the Royal Statistical Society: Series B (Methodological)*.

[28] Chazaud B. (2016). Inflammation during skeletal muscle regeneration and tissue remodeling: application to exercise-induced muscle damage management. *Immunology and Cell Biology*.

[29] Wakahara T., Ema R., Miyamoto N., Kawakami Y. (2017). Inter- and intramuscular differences in training-induced hypertrophy of the quadriceps femoris: association with muscle activation during the first training session. *Clinical Physiology and Functional Imaging*.

[30] Fonseca R. M., Roschel H., Tricoli V. (2014). Changes in exercises are more effective than in loading schemes to improve muscle strength. *The Journal of Strength & Conditioning Research*.

[31] Hakkinen K., Kallinen M., Izquierdo M. (1998). Changes in agonist-antagonist EMG, muscle CSA, and force during strength training in middle-aged and older people. *Journal of Applied Physiology*.

[32] Fortunato A. K., Pontes W. M., de Souza D. M. S. (2018). Strength training session induces important changes on physiological, immunological, and inflammatory biomarkers. *Journal of Immunology Research*.

[33] Quiles J. M., Klemp A., Dolan C. (2020). Impact of resistance training program configuration on the circulating brain-derived neurotrophic factor response. *Applied Physiology, Nutrition, and Metabolism*.

[34] Zhang C., Li Y., Wu Y., Wang L., Wang X., Jie D. (2013). Interleukin-6/signal transducer and activator of transcription 3 (STAT3) pathway is essential for macrophage infiltration and myoblast proliferation during muscle regeneration. *Journal of Biological Chemistry*.

[35] Buford T. W., Cooke M., Willoughby D. (2009). Resistance exercise-induced changes of inflammatory gene expression within human skeletal muscle. *European Journal of Applied Physiology*.

[36] Lambert C. P., Wright N. R., Finck B. N., Villareal D. T. (2008). Exercise but not diet-induced weight loss decreases skeletal muscle inflammatory gene expression in frail obese elderly persons. *Journal of Applied Physiology*.

[37] Hirose L., Nosaka K., Newton M. (2004). Changes in inflammatory mediators following eccentric exercise of the elbow flexors. *Exercise Immunology Review*.

[38] Suzuki K., Nakaji S., Yamada M., Totsuka M., Sato K., Sugawara K. (2002). Systemic inflammatory response to exhaustive exercise. *Cytokine kinetics. Exercise immunology review*.

[39] Akerstrom T., Steensberg A., Keller P., Keller C., Penkowa M., Pedersen B. (2011). Retraction: Exercise induces interleukin-8 expression in human skeletal muscle. *The Journal of Physiology*.

[40] Earp J. E., Newton R. U., Cormie P., Blazevich A. J. (2015). Inhomogeneous quadriceps femoris hypertrophy in response to strength and power training. *Medicine & Science in Sports & Exercise*.

